# Machine Learning Assisted MRI Characterization for Diagnosis of Neonatal Acute Bilirubin Encephalopathy

**DOI:** 10.3389/fneur.2019.01018

**Published:** 2019-10-01

**Authors:** Zhou Liu, Bing Ji, Yuzhong Zhang, Ge Cui, Lijian Liu, Shuai Man, Ling Ding, Xiaofeng Yang, Hui Mao, Liya Wang

**Affiliations:** ^1^Graduate School, Nanchang University School of Medicine, Nanchang, China; ^2^Department of Radiology and Imaging Sciences, Emory University School of Medicine, Atlanta, GA, United States; ^3^Department of Radiology, The People's Hospital of Longhua, Southern Medical University, Shenzhen, China; ^4^Department of Radiation Oncology, Emory University School of Medicine, Atlanta, GA, United States; ^5^Department of Radiology, National Cancer Center/Cancer Hospital & Shenzhen Hospital, Chinese Academy of Medical Sciences and Peking Union Medical College, Shenzhen, China; ^6^Department of Pediatrics, The People's Hospital of Longhua, Southern Medical University, Shenzhen, China

**Keywords:** magnetic resonance imaging, neonate, bilirubin encephalopathy, myelination, machine learning, radiomics

## Abstract

**Background:** The use of magnetic resonance imaging (MRI) in diagnosis of neonatal acute bilirubin encephalopathy (ABE) in newborns has been limited by its difficulty in differentiating confounding image contrast changes associated with normal myelination. This study aims to demonstrate the feasibility of building a machine learning prediction model based on radiomics features derived from MRI to better characterize and distinguish ABE from normal myelination.

**Methods:** In this retrospective study, we included 32 neonates with clinically confirmed ABE and 29 age-matched controls with normal myelination. Radiomics features were extracted from the manually segmented region of interest (ROI) on T1-weighted spin echo images, followed by the feature selection using two-sample independent *t*-test, least absolute shrinkage and selection operator (Lasso) regression, and Pearson's correlation matrix. Additional feature quantifying the relative mean intensity of ROI was defined and calculated. A prediction model based on the selected features was built to classify ABE and normal myelination using multiple machine learning classifiers and a leave-one-out cross-validation scheme. Receiver operating characteristics (ROC) analysis was used to evaluate the prediction performance with the area under the curve (AUC) and feature importance ranked based on the Fisher score.

**Results:** Among 1319 radiomics features, one radiologist-defined intensity-based feature and 12 texture features were selected as the most discriminative features. Based on these features, decision trees had the best classification performance with the largest AUC of 0.946, followed by support vector machine (SVM), tree-bagger, logistic regression, Naïve Bayes, discriminant analysis, and k-nearest neighborhood (KNN), which have an AUC of 0.931, 0.925, 0.905, 0.891, 0.883, and 0.817, respectively. The relative mean intensity outperformed other 12 texture features in differentiating ABE from controls.

**Conclusions:** The results from this study demonstrated a new strategy of characterizing ABE-induced intensity and morphological changes in MRI, which are difficult to be recognized, interpreted, or quantified by the routine experience and visual-based reading strategy. With more quantitative and objective measurements, the reported machine learning assisted radiomics features-based approach can improve the diagnosis and support clinical decision-making.

## Introduction

Neonatal jaundice is one of the most prominent clinical concerns during the neonatal period. It is mainly caused by the accumulation of neurotoxic unconjugated bilirubin from the breakdown of old red blood cells that cannot be cleared effectively by newborns (1). Based on a nationwide survey on hospitalized neonates in China, 49.1% developed various degrees of neonatal jaundice and 8–9% developed severe hyperbilirubinemia with as high as 0.9% (357/4,141,535) ended up developing bilirubin encephalopathy due to lack of appropriate diagnosis and prediction of its development or delayed treatment (2). Kernicterus, the brain damage specifically caused by hyperbilirubinemia, is characterized by the intense yellow staining of bilirubin at some specific regions of the brain, which is consistent across the term, preterm, and rare adult with kernicterus (3). Before kernicterus (chronic bilirubin encephalopathy, CBE), a permanent neurological sequela induced by bilirubin toxicity, hyperbilirubinemia and acute bilirubin encephalopathy (ABE) can be reversed with safe and effective treatments (4). Therefore, identifying neonates with a high risk of ABE early to apply the treatment timely is the key to minimize the incidence of permanent bilirubin-induced neurological dysfunction (BIND) and kernicterus (5). However, early detection of jaundiced neonates with the risk of brain damage in the acute stage is challenging in the current clinical practice.

The evaluation of neonatal jaundice is typically done with the standard clinical laboratory test by measuring the total serum bilirubin (TSB) concentration. Because it is not a direct measurement of the actual bilirubin level in the brain, TSB measurement leads to considerably high false-positive and false-negative rates when used as a predictor of ABE (6). Including other serum parameters, such as unconjugated or “free” bilirubin, albumin level, and bilirubin-albumin binding capacity, does not significantly improve the overall prediction power (7, 8). As for clinical manifestations, the early neurological symptoms induced by ABE could be absent, subtle, or non-specific in most cases (9). When a clinical sign of the classic tetrad syndrome caused by CBE appears, the bilirubin toxicity-induced neural injuries have already become permanent and irreversible. Moreover, several comorbidities, such as hemolytic diseases, prematurity, asphyxia, or infection, can all pose neonates with jaundice to a higher risk of ABE, which further compromise the predictive performance of serum measurements and clinical manifestations (10). Therefore, there is a great need in non-invasive and direct detection of bilirubin-induced subtle change in the infant brain to assess the risk of brain damage in ABE.

Magnetic resonance imaging (MRI), as a non-radiation and non-invasive imaging technique, offers superb resolution and soft tissue contrast for visualizing brain structures and abnormalities. Thus, it is well suited for safe and direct imaging of the neonatal brain affected by bilirubin toxicity. It not only can provide brain region-specific evidence for ABE but also enables to exclude hypoxic–ischemic encephalopathy, the most common neonatal encephalopathy (11). During the days to weeks of ABE, MRI shows “classic” T_1_-signal hyperintensity in various degrees on T_1_-weighted spin echo images in the globus pallidus and subthalamic nuclei, hippocampus, and cerebellum in the neonatal brain accompanied by an unremarkable or subtle signal increase on T_2_-weighted images (12–14). However, normal myelination in newborns within the same age span can also lead to slightly increased signal intensity on T_1_-weighted images in the same regions similar to the bilirubin-induced hyperintensity in the neonates with ABE (15). Therefore, it is difficult for the conventional signal intensity change-based reading strategy that is commonly used in radiology reading and evaluations to yield accurate diagnosis.

With emerging radiomics and machine learning assisted image analysis, various graphical features, especially those difficult to be recognized by radiologists, can be identified and extracted to generate a large set of data to further correlate with pathological (16), genomic (17), molecular (18), and clinical outcome (19) information. Thus, radiomics analysis can expand the capability of characterizing disease-induced image abnormalities and the underlying pathophysiology in much greater details with parametric variables and high-throughput quantitative measurements to improve the accuracy of diagnosis and predicting prognosis (20, 21).

Herein, we report an initial work of applying the radiomics-based machine learning approach to identify an ABE-specific radiomics pattern based on MRI contrast changes on T_1_-weighted spin echo images associated with the bilirubin deposition in neonatal brains and its induced change in tissue properties. The distinct radiomics features were found and used to differentiate ABE from normal myelination.

## Materials and Methods

### Study Subjects

This retrospective study was approved by the Institutional Review Board and carried out in accordance with the Declaration of Helsinki with the written informed consent waived from legal custodians of all subjects for this study. However, as a part of clinical routine procedure, the written informed consent for MRI examinations was obtained from their legal custodians before MRI examinations. We selected 61 neonates based on reviewing their medical records, including 32 ABE neonates with a mean after-birth age of 6.8 ± 3.5 days and 29 age-matched controls (11.6 ± 6.1 days), who had MRI examinations during their hospitalization. For more accurate indication of maturity of the newborns, we used the average equivalent age, defined as gestational age + age after birth. The average equivalent age is 276.1 ± 10.4 days for 32 neonates with suspected ABE and 262.1 ± 22.3 days for 29 age-matched controls, respectively. To minimize the potential age-related factor, we only included those neonates with the age within 3 weeks after birth, during which ABE usually develops. All 32 ABE cases were clinically confirmed based on their medical records. To standardize inclusion criteria, we set stringent inclusion criteria by collecting supportive information for diagnosing ABE from the medical records of all subjects. All ABE positive cases met at least two of three clinical diagnosis criteria, including (1) severe hyperbilirubinemia (peak total serum bilirubin ≥20 mg/dl or 342 μmol/L); (2) positive radiological findings suggestive of ABE; (3) at least one of the ABE-related clinical symptoms with bilirubin-induced neurologic dysfunction (BIND) score ≥ 1 point in which 1, 2, or 3 points were assigned to mild, moderate, or severe symptoms based on the severity of the crying pattern defined for neonates, behavior and mental status, and muscle tone for a total 9 points (22). Neonates with any history of neurological abnormalities caused by perinatal asphyxia, hypoxia–ischemia, intrauterine infection, chromosomal disease, hereditary mitochondrial metabolic disease, carbon monoxide poisoning, and hypermagnesemia and other related diseases were excluded. T_1_-weighted images from each case were evaluated for motion artifacts that may affect the image analysis. All cases included have satisfactory image quality for further analysis.

All clinically confirmed ABE cases had a peak TSB level ≥20 mg/dl during hospitalization. With laboratory test and clinical manifestations available for all 32 positive ABE cases during interpretation, previous radiology reports showed that radiologists diagnosed 5 out of 32 cases matching the typical imaging findings of ABE, 18 cases highly likely to have developed ABE, 7 cases likely but indecisive to have ABE, and 2 cases unlikely to have ABE, but they cannot exclude the possibility in reference to the clinical information. Of 32 ABE neonates, 26 developed explicit ABE-specific clinical symptoms with BIND scores ranging from 1 to 6, while 6 ABE cases developed ABE-non-specific clinical symptoms based on the available medical records. In contrast, 29 age-matched control cases were negative in all three criteria mentioned above.

### MRI Acquisition

All images were acquired from a 1.5-T whole-body MRI scanner (Achiva, Philips Healthcare, Best, the Netherlands) using a routine clinical brain MRI protocol. The protocol included the following imaging sequences: T_1_-weighted spin-echo imaging in the axial and sagittal directions, and T_2_-weighted fast spin echo imaging, T_2_-weighted fluid-attenuated inversion recovery (FLAIR) imaging, and diffusion-weighted imaging (DWI) in the axial direction. Because the characteristic image appearance for ABE is elevated signal intensity in globus pallidus and subthalamic nuclei on T_1_-weighted spin echo images, we only focused on analyzing T_1_-weighted spin echo images in this study. The imaging parameters for T_1_-weighted spin echo imaging included the following: echo time (TE) of 17 ms, repetition time (TR) of 600 ms, flip angle of 69°, field of view (FOV) of 150 × 133 × 79 mm, and 18 slices with a slice thickness of 4 mm to cover the whole brain.

### Image Preprocess

Images from all cases were visually examined first by the radiologists (ZL and LW) for the image quality and artifacts. We then used the Smallest Univalue Segment Assimilating Nucleus (SUSAN) technique (FSL v5.0, FMRIB, Oxford, UK), a filtering technique that preserves the structures depicted in an image by only averaging a central voxel with neighboring voxels that have similar intensities (23), to reduce noise. FMRIB Linear Image Registration Tool (FLIRT) (FSL v5.0, FMRIB, Oxford, UK) (24) was then used to align the images to correct any motion artifacts before any analysis. Then, a histogram stretching algorithm was used for normalizing the image intensity of all images (25).

### Lesion Identification and Segmentation

**Figure 1:**
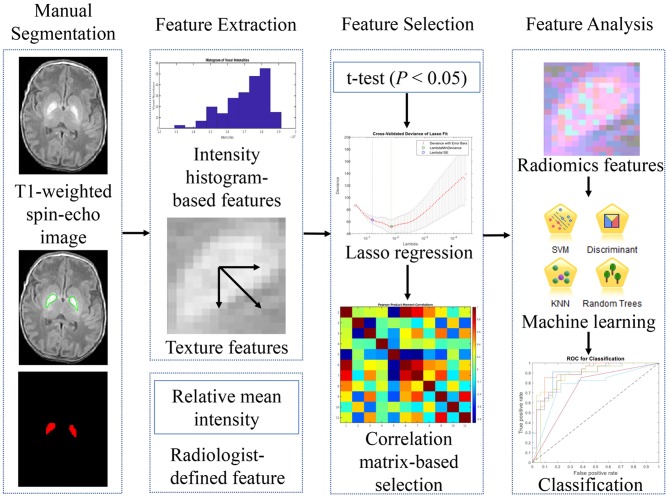
The workflow for the image processing and analyses in the study.

**Figure 2:**
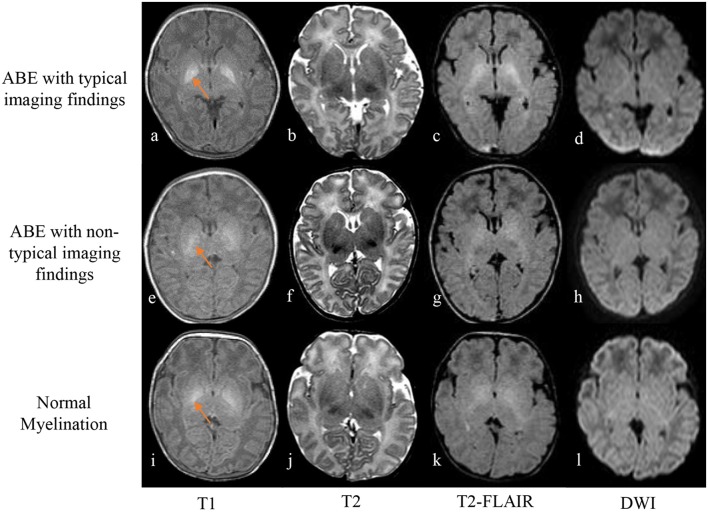
Axial T_1_-weighted spin echo image **(a)** shows strikingly hyperintense signal in the globus pallidus with well-defined boundary in a neonate of 10 days old with ABE **(a–d)**, while T_2_-weighted fast spin echo image **(b)** and FLAIR image **(c)** show slightly and moderately high signal intensity, compared with the signal intensity of the surrounding basal ganglia region, respectively. No abnormal signal intensity was detected on DWI **(d)**. However, in cases of a 4-day-old neonate with radiologically untypical ABE **(e–h)** and a case of 9-day-old ABE-alike neonate with normal myelination **(i–l)**, imaging findings include hazy slight hyperintensity on T_1_-weighted image **(e,i)**, iso-intensity on T_2_-weighted image **(f,j)**, slightly high signal intensity on T_2_-weighted FLAIR images **(g,k)**, and no obvious abnormal signal intensity on DWI **(h,l)** in the region of globus pallidus with referencing the signal intensity of the surrounding region of basal ganglia.

The pipeline of the feature identification and analysis processes is summarized in Figure 1. For the current study, we only focused on the abnormalities in globus pallidus, which is one of the earliest and most sensitive regions affected by the bilirubin toxicity in ABE (26). An abnormal appearance of increased signal intensity on T_1_-weighted spin echo images in the region is shown in Figure 2, allowing for extracting globus pallidus from T_1_-weighted images as the region of interest (ROI). In the ABE case with characteristic imaging findings, the bilateral globus pallidus has a sharp contrast with a well-defined margin for manual segmentation. One radiologist (ZL) with more than 5 years of experience in neuroimaging manually contoured the structure of globus pallidus using open source Imaging Biomarker Explorer Software (IBEX software, MD Anderson Cancer Center, Houston, Texas, US) (27). When the contour of globus pallidus was not well-defined, we referenced the cross-sectional gross anatomy of globus pallidus and also used the silhouette of an ABE case with typical image characteristics as the reference (Figure 2). Instead of segmenting globus pallidus independently in different groups, we referenced the clear silhouette of ABE cases with typical imaging findings of ABE in order to ensure that the segmented globus pallidi were comparable in shape between ABE cases and controls. Similarly, we manually delineated the boundary of globus pallidus in control cases based on the cross-sectional neuroanatomy and the corresponding regions of ABE cases with well-defined globus pallidus. In total, three continuous slices containing globus pallidus on T_1_-weighted images for each neonate were chosen. After initial segmentation, each ROI was reviewed and adjusted by two radiologists (LW and YZ) with more than 25 years of experience in neuroradiology, with any discrepancy resolved through discussion.

### Feature Extraction

Once the segmented globus pallidus was obtained, 1,318 radiomics features from ROIs were extracted using IBEX software. All of the extracted features were then grouped into two main categories and nine subcategories (27), including the first-order statistics (intensity-histogram-based features): (1) intensity histogram gauss fit-based features; (2) histogram gradient orientation-based features; (3) intensity histogram-based features; (4) intensity direct-based features and higher-order statistics (texture features): (5) gray level co-occurrence matrix-based features [GLCM, 2-Dimensions (2-D)]; (6) GLCM (3-D); (7) gray level run length matrix-based features (GLRLM); (8) neighborhood gray-tone difference matrix features (NGTDM, 2-D); and (9) NGTDM (3-D) (28).

To follow the conventional reading strategy and to quantify the average intensity level of globus pallidus, we added a “radiologist-defined feature,” i.e., the relative mean intensity of globus pallidus, which corresponds to the average intensity of segmented globus pallidus in the second slice of the three chosen slices normalized by the average intensity of cerebrospinal fluid (CSF) in the segmented region of lateral ventricle of the same slice. Then, two histograms based on the distribution of the relative mean intensity of ABE cases and control cases were generated and curve-fitted by non-linear polynomial regression using MATLAB (2018b, Mathworks, USA).

### Feature Selection

To improve the accuracy and efficiency of classifications, irrelevant and redundant features were identified and excluded. We used three feature selection methods sequentially to select informative radiomics features from 1,318 graphic features obtained using IBEX software. Firstly, a two-sample *t*-test was used to select those features with statistically significant difference between ABE cases and control cases (*P* < 0.05). To increase the model interpretability and reduce overfitting, the least absolute shrinkage and selection operator (Lasso) regression algorithm (29) was then applied to the remaining features to further reduce irrelevant features. Those features yielding to the lambda (λ) value (a tuning parameter) with minimal deviance were chosen as the most informative features.

### Feature Correlation

To follow the principle that good features are highly correlated with the predictive target, but remain to be independent to each other (30), we evaluated the correlation between each pair of the selected features. A correlation-matrix map was generated based on the correlation between each chosen feature to illustrate and determine the redundancy. In correlation-based feature elimination, we did not remove all correlated features like previous studies (31, 32). Instead, we only removed those features with an *r*-value of 1 or −1, termed as linearly correlated features, which usually are calculated using the same formula, but different directions and offsets based on the generated matrix. Then, one representative feature in each group of linearly correlated features was used as the discriminative feature for further analysis, since this feature can represent the other linearly correlated features within the same group. The remaining features (−1 < *r* < 1) were put into the group of relatively independent features.

### Classification

The features chosen for classifying ABE and normal myelination conditions included the selected most discriminative features and the radiologist-defined feature. Based on multiple classification algorithms, including logistic regression, discriminant analysis, k-nearest neighborhood (KNN), Naïve Bayes, support vector machine (SVM), decision trees, and ensemble tree-bagger (33), and the chosen features, a prediction model was built to differentiate ABE from normal myelination with a leave-one-out cross-validation scheme used to split the data into training set and testing set randomly. To compare the overall performance of each classifier, the value of the area under the curve (AUC) was calculated based on receiver operating characteristics (ROC) analysis. Also, the accuracy was obtained from the best cutoff point in the ROC curve for each classifier. Then, features were ranked based on their importance contributing to the classification performance using the Fisher score, an independent filter model aiming to not interact with the bias of a classification learning algorithm (34).

### Statistical Analysis

MATLAB (2018b, Mathworks, USA) was used to perform all the statistical analysis, which included calculating the relative mean intensity of the segmented globus pallidus, generating the histogram of relative mean intensity of globus pallidus, feature selection using two-sample *t*-test, Lasso regression algorithm, and correlation-matrix heat map generated using Pearson's correlation, classification using multiple classification algorithms with ROC analysis, and features ranking based on Fisher score. Nonparametric Mood's median test was used to determine whether the medians of the two independent groups (ABE group vs. normal myelination group) from which two samples are drawn are identical, with *P* < 0.05 indicating statistically significant difference. Then, two-sample independent *t*-test was used to compare clinical variables, such as birth weight and different kinds of bilirubin level, with *P* < 0.05 indicating statistically significant difference.

## Results

### Clinical Findings and Subject Characteristics

In 61 neonates included in this study, we found no statistically significant difference in the average equivalent age (*P* = 0.063) between neonates with ABE (*n* = 32, 276.1 ± 10.4 days) and age-matched controls with normal myelination (*n* = 29, 262.1 ± 22.3 days). It should be noted that controls had more preterm neonates (16/29 vs. 5/32) and slightly lower birth weight (2686.7 ± 918.2 vs. 3027.5 ± 404.6 g) but with no statistical difference (*P* = 0.107). The neonates with ABE had a much higher mean bilirubin level than did controls as shown by the transcutaneous bilirubin level (25.3 ± 5.5 vs. 10.9 ± 2.3 mg/dl), peak TSB (500.1 ± 78.8 vs. 139.3 ± 58.3 μmol/L), and unconjugated serum bilirubin (482.8 ± 73.4 vs. 127.4 ± 58.3 μmol/L) (all *P* < 0.00001). Demographic information is summarized in Table 1.

**Table 1:** Clinical characteristics of neonates with ABE and control neonates.

	**Neonates with ABE**	**Control neonates**	***P-*value**
Gender (male/female)	15/17	19/10	–
Equivalent age (days)	276.1 ± 10.4	262.1 ± 22.3	0.063
Term/preterm	27/5	13/16	–
Birth weight (g)	3027.9 ± 404.6	2686.7 ± 918.2 (2 NA)	0.106
Transcutaneous bilirubin level (mg/dl)	25.3 ± 5.5 (1 NA)	10.9 ± 2.3 (11 NA)	<0.00001
Peak total serum bilirubin (pTSB) (μmol/L)	500.1 ± 78.8	139.3 ± 58.3 (2 NA)	<0.00001
Unconjugated serum bilirubin (μmol/L)	482.8 ± 73.4	127.4 ± 58.3 (2 NA)	<0.00001

*NA, not available; equivalent age = gestational age + age after birth*.

Figure 2 shows images of an ABE case with characteristic imaging contrast change in the affected region, an ABE case with ambiguous imaging findings, and a control case with normal myelination. The neonates with radiologically typical ABE (2/32) exhibited bilateral symmetrical pronounced hyperintensity in the globus pallidus on T_1_-weighted spin echo images with a well-defined boundary and no obvious signal abnormalities on T_2_-weighted spin echo, FLAIR, and DWI (Figures 2a–d). However, most ABE cases (30/32) did not present such sharp contrast on T_1_-weighted spin echo images in the region of globus pallidus with a well-defined boundary but showed varying degrees of increased signal intensity compared to the signal intensity of the surrounding structures of basal ganglia (Figures 2e–h). All the controls also showed slight to moderate elevation of signal intensity in globus pallidus on T_1_-weighted spin echo images varied in different degrees (Figures 2i–l), consistent with the signal intensity change due to normal myelination in newborns. Such a pattern of signal-intensity increase mimics MRI contrast appearance of a radiologically atypical ABE. For T_2_-weighted spin echo images, only 3 ABE cases (3/32) showed slightly high signal intensity in the region of globus pallidus in contrast to the signal intensity of the surrounding basal ganglia region (Figure 2b), and no controls showed any obvious signal changes (Figure 2j). Interestingly, all the ABE cases and controls with normal myelination showed slightly high signal intensity on T_2_-weighted FLAIR images in comparison to the signal intensity of surrounding basal ganglia region (Figures 2c,g,k), suggesting that FLAIR sequence may not be a proper imaging method for differentiating ABE from normal myelination. For DWI, no apparent abnormal signal intensity was found in both ABE and control cases, suggesting no substantial effect from ABE and normal myelination on the diffusion properties of the tissue at this point.

### Radiomics Features

**Figure 3:**
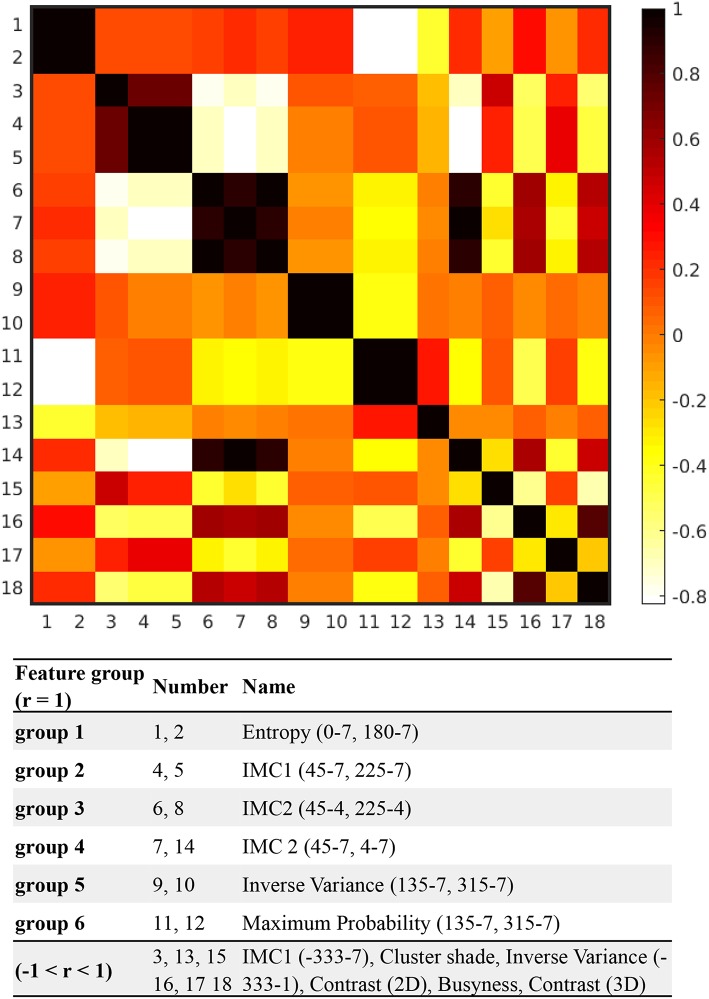
The correlation-matrix heat map based on the correlation between each feature pair of the selected 18 features. All 18 features were calculated with direction of 0, 45, 90, 135, 180, 225, 270, and 315°, and offset of 1, 4, and 7, respectively. For instance, based on the matrix generated from the segmented globus pallidus, entropy (0°-7) was calculated with direction = 0° and offset = 7, while maximum probability (135°-7) indicates that maximum probability was calculated with direction = 135° and offset = 7.

**Figure 4:**
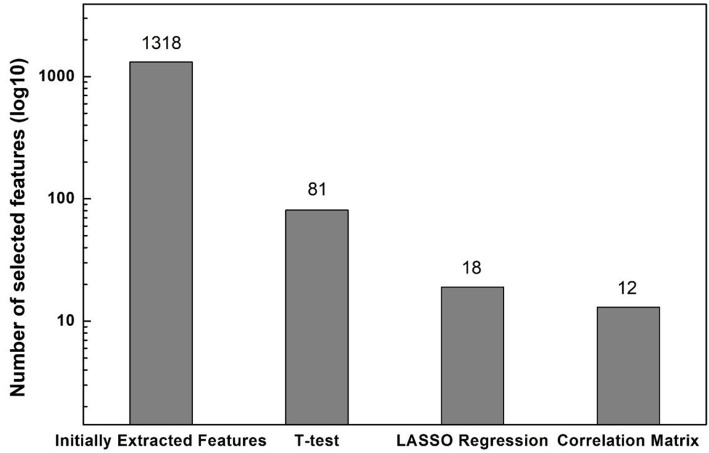
Numbers of features selected after each feature selection method performed sequentially.

The feature extraction program automatically identified 1,318 features from ROIs on T_1_-weighted spin echo images. Among these, 81 features with statistically significant difference between ABE cases and controls (*P* < 0.05) were selected using two-sample *t*-test. After the Lasso regression algorithm was applied for further feature reduction, 18 features were chosen based on the corresponding lambda (λ) value with minimal deviance (Figure S1). Figure 3 shows the correlation between each pair of these 18 features, presented as a heat map of correlation matrix. We found six non-redundant features (−1 < *r* < 1), with six pairs of linearly correlated features (*r* = 1) in the remaining 12 features. After the correlation-matrix-based feature selection, one representative feature for each linearly correlated feature group (*n* = 6) and six relatively independent features were chosen for further analysis (more details in Figure 3). The process of the feature selection is plotted in Figure 4 with more detailed information summarized in Table S1 with descriptions of 12 selected features provided in Table S2.

### Classifications

**Figure 5:**
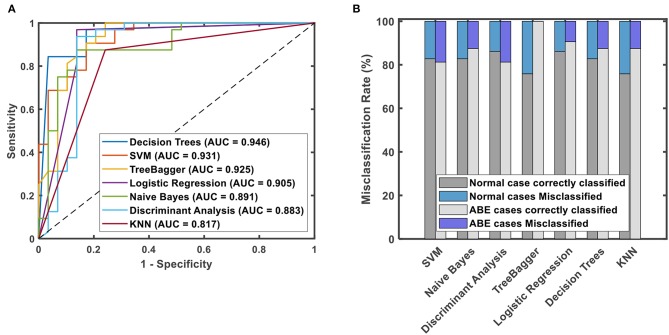
ROC curves with values of AUC for different classification methods using 13 discriminative features **(A)** and misclassification rates for normal cases and ABE cases for different prediction models **(B)**.

Based on the 12 selected features and one radiologist-defined feature, decision trees had the best classification performance with an AUC of 0.946, followed by SVM, tree-bagger, logistic regression, Naïve Bayes, discriminant analysis, and KNN, which have an AUC of 0.931, 0.925, 0.905, 0.891, 0.883, and 0.817, respectively, as shown in Figure 5A. On the other hand, when using the misclassification rate to evaluate the accuracy of discriminating ABE neonates and controls, the logistic regression algorithm and tree-bagger performed better than others with 9.38, 0% for misclassifying ABE and 13.79, 24.14% for misclassifying the control condition, respectively. Detailed comparisons are presented in Figure 5B. As a whole, both logistic regression algorithm and tree-bagger had the highest accuracy of 88.5% to differentiate ABE from controls compared to other machine learning classifiers.

### Feature Ranking and Contribution

**Figure 6:**
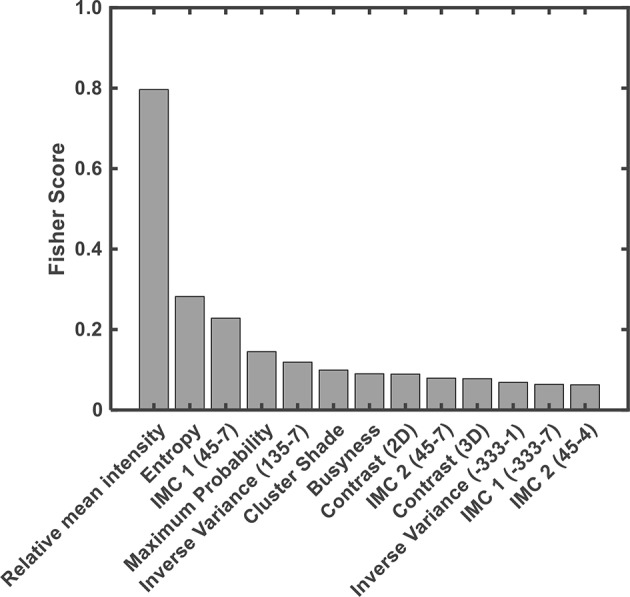
Contributions of features in discriminating ABE and controls were ranked based on their Fisher score. IMC 1 & 2 = information measure of correlation 1 and 2.

**Figure 7:**
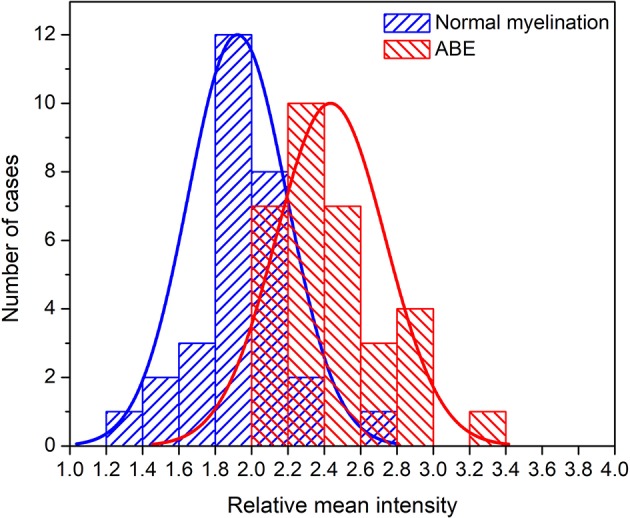
Histograms of the relative mean intensity distribution plots from the groups of ABE and control with normal myelination.

Thirteen features used for classification were ranked using the Fisher score (Figure 6) according to their importance in discriminating ABE from the normal myelination. Among them, the relative mean intensity, which reflects the overall brightness of segmented globus pallidus, was the most discriminative feature. It is significantly higher in ABE cases than that in controls (*P* < 0.0001). Importantly, the results from radiomics analysis of image features provided insight into the inherited challenges of using the conventional signal intensity-focused radiology reading strategy to diagnose ABE. Histograms of the relative mean intensity from the segmented globus pallidus in the ABE and control groups shown in Figure 7 revealed the significant overlap of similar image contrast of ABE and normal myelination conditions that contribute to the difficulty of distinguishing ABE from normal myelination, if it is simply based on the signal intensity change in the images.

In addition, nine different texture features in the GLCM category had significant contributions to the discrimination of ABE from normal myelination. Finally, three other texture features selected in the category of NGTDM, including contrast computed from 2D to 3D images, respectively, and busyness were found to be useful in the classification as they revealed intra-lesion spatial neighbor intensity difference of abnormalities. However, no first-order statistical feature (i.e., intensity histogram-based feature) and feature within the category of GLRLM were found as distinct features based on the current criteria used in this study.

## Discussion

Although MRI has been increasingly used to investigate the neuropathology induced by ABE in the neonatal clinical settings, the conventional reading strategy solely based on overall intensity alteration of the globus pallidus on T_1_-weighted images is not sufficiently effective and accurate. The current study applied a radiomics-based machine learning approach to extract specific image features to discriminate neonates with ABE from controls with normal myelination. The results suggested that this approach improved the characterization of abnormalities and thus achieved a better classification of these two conditions.

Clinically, most ABE cases do not show characteristic signal abnormalities in MRI and thus are not readily distinguishable from normal myelination. As shown in this study, only two cases with ABE showed typical striking hyperintensity in the region of globus pallidus, while other cases of ABE have various degrees of hyperintense contrast that overlap with the signal changes from normal myelination. In clinical routine, the common reading strategy of radiologists focuses on identifying the relatively higher signal intensity induced by ABE than that of normal myelination on T_1_-weighted image, since usually, signal abnormality tends to vary from moderately high to very high for ABE and slightly high to moderately high for normal myelination based on poorly quantitative visual impression. However, depending on the experience and expertise level of radiologists, such a subjective judgment is susceptible to inter- and intra-observer variability in interpreting ABE. Worth noting, the radiologist-defined feature, the relative mean intensity of the segmented globus pallidus outperformed 12 selected texture features as the most discriminative feature to differentiate ABE from normal myelination, suggesting the robustness of the conventional intensity-based reading strategy used by the radiologists who have developed sufficient experience in recognizing subtle intensity changes and differences caused by the disease. However, compared to the experience-dependent traditional visual-based pattern recognition, using computational algorithms to extract fine graphical features and descriptors on changes in image intensity and contrast in a more quantitative manner can eliminate the inter- and intra-observer subjective variability in interpreting ABE.

The study also showed that additional texture features that are not readily recognized, described, or quantified by radiologists in the clinical routine reading can be identified by computational methods, allowing for using more quantitative parameters to better characterize the lesion and further enhance the discrimination of these two conditions. In our study, we found that three NGTDM features and nine GCLM features contributed to separating ABE from normal myelination. NGTDM features revealed the pattern of intra-lesion spatial intensity difference (28) of ABE associated with tissue relaxation time change induced by bilirubin toxicity. In contrast, GCLM features, which represent the spatial distribution of various gray-level combinations, reveals the regional heterogeneity of the affected tissue (35). The high accuracy in distinguishing ABE from normal myelination based on these morphological heterogeneity-associated texture features demonstrated the feasibility of utilizing additional texture features to detect, describe, and quantify the morphological heterogeneity of the globus pallidus induced by bilirubin accumulation. Importantly, it should be noted that this approach can be potentially expanded to diagnosing other types of neonatal encephalopathy that is diagnosed only based on signal intensity change.

ABE-positive cases included in the study were clinically confirmed with either positive serum indicators, and (or) positive imaging findings, and (or) neurological or behavioral symptoms. However, BIND scores cannot be obtained from some ABE cases due to either overlooked subtle neurological or behavioral symptoms or incomplete medical records in this retrospective study. In the future, a prospective study performing BIND score evaluation for any neonates with suspicious ABE is needed. The current proof-of-concept study only included a limited number of ABE subjects due to that MRI has yet to become a clinical standard practice for evaluating ABE and remaining technical challenges in neonatal MRI. Therefore, we did not attempt to follow these patients and further evaluate the possibility of using radiomics features to sub-classify these ABE cases to determine the severity of the conditions and difference between preterm and term neonates. Besides, we have applied multiple cross-validation methods, such as 10-, 5-fold, and leave-one-out method to reduce overfitting. Finally, we optioned to use leave-one-out method for its resulting relatively lower accuracy and AUC, considering minimizing the possibility of overfitting given a relatively small sample size. It is anticipated that training machine learning algorithms on a large cohort with larger sample size and more heterogeneous data acquired on an MRI scanner with higher magnetic field and testing them on an independent cohort should further validate the robustness of this approach to generalize a clinical feasible predictive model.

Another limitation of the current study is that we used manual contouring for segmentation of ROI, which is prone to inter-observer variability. We ensured to minimize the segmentation inaccuracy by referencing the cross-sectional gross anatomy and the ROIs determined in the ABE cases with typical imaging findings via double-blinded reading by the experienced radiologists. Although manual delineation is time-consuming and subject to introducing the bias from interpreters, it is currently considered as a “gold standard” for segmentation (36). A semiautomatic method, combining expert-based manual delineation and automatic segmentation algorithm, might improve the segmentation accuracy and thus further improve the accuracy of downstream classification in the future. Furthermore, the current study only focused on extracting radiomics features from T_1_-weighted images, attempting to follow the conventional reading strategy of interpreting ABE for comparison. It is expected that the classification performance of the reported approach will be further improved by incorporating features extracted from multi-parametric MRI, including those features associated with tissue microenvironment alterations, such as relaxation time change (T_1_-mapping), cellularity (DWI), integrity and maturity of white matter tract (diffusion tensor imaging), and metabolism (magnetic resonance spectroscopy).

## Conclusion

The current study demonstrated the feasibility of using a radiomics-based machine learning approach to analyze overlapped hyperintense signal patterns of globus pallidus on T_1_-weighted spin echo images between neonates with ABE and normal myelination to improve the differentiation of these two conditions. Compared to the experience-dependent visual-based conventional reading strategy, incorporating radiomics features improved the lesion characterizations with more descriptors and more quantitative and objective measurements for ABE-induced intensity change and morphological heterogeneity. The results support the potential utility of such an approach to assist the clinical prediction on the risk and development of neurological damages in neonates with hyperbilirubinemia.

## Data Availability Statement

The datasets generated for this study are available on request to the corresponding author.

## Ethics Statement

This retrospective study is approved by the Institutional Review Board of the People's Hospital of Longhua, Shenzhen China. The study was carried out in accordance with the Declaration of Helsinki with written informed consent waived from the custodians of all subjects.

## Author Contributions

LW and HM contributed to project idea and supervision and to manuscript revision, and maintained integrity of this manuscript. ZL implemented the whole study, analyzed the data, and drafted the manuscript. BJ and YZ contributed to statistical analysis and reviewed the manuscript. LD, SM, and LL collected the raw data. GC and XY provided technical support. All authors had reviewed this manuscript critically and approved its final submission.

### Conflict of Interest

The authors declare that the research was conducted in the absence of any commercial or financial relationships that could be construed as a potential conflict of interest.

## Abbreviations

CBE: chronic bilirubin encephalopathy
ABE: acute bilirubin encephalopathy
BIND: bilirubin-induced neurological dysfunction
FLAIR: fluid-attenuated inversion recovery
DWI: diffusion-weighted imaging
ADC: apparent diffusion coefficients
FOV: field of view
TE: echo time
TR: repetition time
ROI: region of interest
Lasso: least absolute shrinkage and selection operator
KNN: k-nearest neighborhood
SVM: support vector machine
AUC: area under the curve
ROC: receiver operating characteristics
GLCM: gray level co-occurrence matrix
GLRLM, gray level run length matrix, NGTDM: neighborhood gray-tone difference matrix
IMC 1 and 2: information measure of correlation 1 and 2.
