# Supplementary material for: Machine Learning Assisted MRI Characterization for Diagnosis of Neonatal Acute Bilirubin Encephalopathy
Source: Front Neurol. 2019 Oct 1;10:1018. doi: 10.3389/fneur.2019.01018 (PMC6779823; doi:10.3389/fneur.2019.01018)
Supplement: Supplementary file 1 [file Table_1.DOCX]

**Table S1**: Numbers and types of features selected in feature selection steps

| Feature Category | Number of extracted features | | Features selected after t-test | Features selected after Lasso regression | Features selected after correlation matrix |
| --- | --- | --- | --- | --- | --- |
| Histogram of Oriented Gradient | 46 | | 0 | 0 | 0 |
| GLCM 2D^a^ | 592 | | 45 | 12 | 7 |
| GLCM 3D^b^ | 527 | | 29 | 3 | 2 |
| GLRLM 2D^a^ | 33 | | 0 | 0 | 0 |
| Intensity Direct | 56 | | 0 | 0 | 0 |
| Intensity Histogram | 49 | | 0 | 0 | 0 |
| Intensity-Histogram-Gauss Fit | 5 | | 1 | 0 | 0 |
| NGTDM 2D^a^ | 5 | | 2 | 1 | 1 |
| NGTDM 3D^b^ | 5 | | 4 | 2 | 2 |
| Total | | 1318 | 81 | 18 | 12 |

GLCM = gray level co-occurrence matrix, GLRLM = gray level run length matrix, NGTDM = neighborhood gray-tone difference matrix. 2D^a^: feature is computed from all 2-Dimensional image slices, while 3D^b^: feature is computed from 3-Dimensional image matrix.

**Table S2**: Descriptions of 12 selected radiomics features

| **Category** | **Feature Name** | **Description** |
| --- | --- | --- |
| GLCM | Entropy (0-7^a^) | Entropy measures the randomness of intensity distribution (disorder or complexity of an image), with lower values for smooth images than for a coarse image. |
|  | IMC 1 (45-7^a^&-333-7^a^)  IMC 2 (45-7^a^& 45-4^a^) | Both IMC1 and IMC2 assess the correlation between the probability distributions (quantifying the complexity of the texture), using mutual information. |
|  | Inverse Variance  (135-7^a^&-333-1^b^) | Inverse Variance measures the homogeneity of an image. |
|  | Maximum Probability (135-7^a^) | Maximum probability is the occurrence of the most predominant pair of neighboring intensity values. |
|  | Cluster Shade (9-1^b^) | Cluster shade is a measure of the skewness of the matrix and is believed to gauge the perceptual concepts of uniformity. |
| NGTDM | Contrast^a&b^ | Contrast is a measure of the spatial intensity change. But it is also dependent on the overall gray level dynamic range. |
|  | Busyness^b^ | Busyness is a measure of the change from a pixel to its neighbor. A high value for busyness indicates rapid changes of intensity between pixels and its neighborhood. |

^a^Feature is computed from all 2D image slices. ^b^Feature is computed from 3D image matrix. For the meaning of 0-7, entropy (0-7) means entropy is calculated with direction = 0 º and offset = 7 based on the matrix generated from segmented lesion. IMC 1 & 2 = Information Measure of Correlation 1 & 2; GLCM = gray level co-occurrence matrix; NGTDM = neighborhood gray-tone difference matrix.


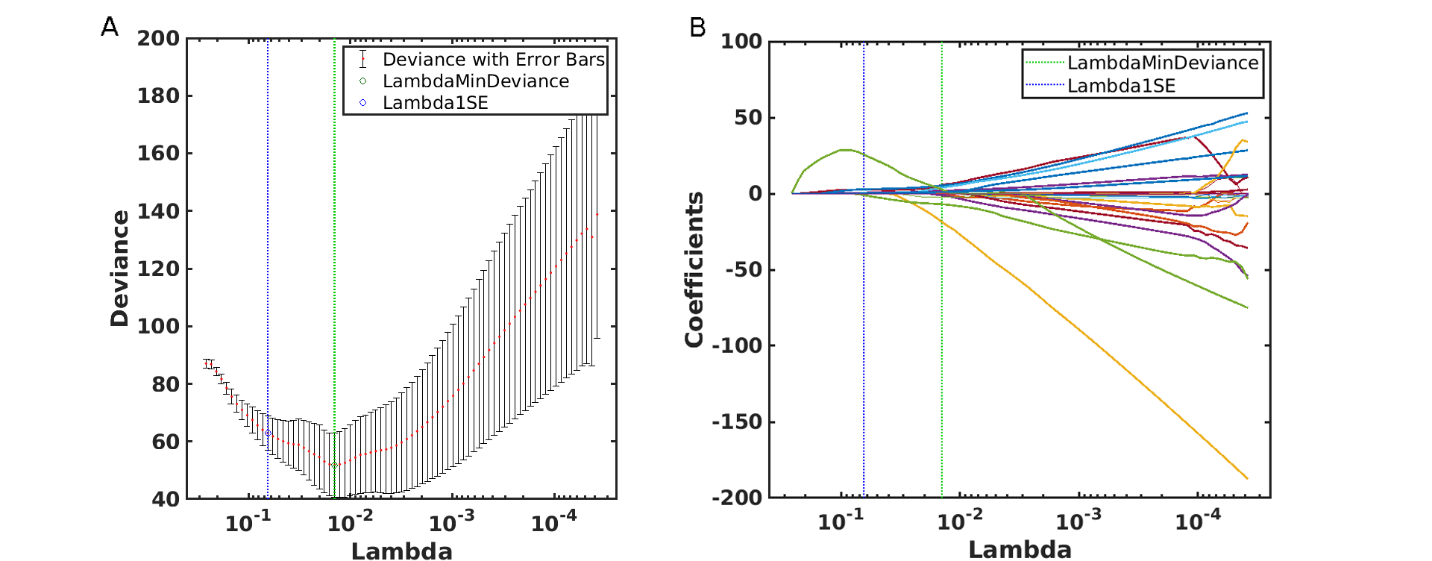


**Figure S1**: Feature selection using Lasso regression model. (A): The dotted green line and blue line indicate the best lambda value based on the minimal deviance and its one standard error, respectively. The minimal deviance corresponded to the optimal number of features. (B): The Lasso coefficient profiles of the 81 features. The dotted green line corresponded to 18 features with nonzero coefficients indicating the best lambda value based on the minimal deviance.
